# Effects of behaviour change communication on hypertension and diabetes related knowledge, attitude and practices in Imo and Kaduna States: a quasi-experimental study

**DOI:** 10.1186/s12889-022-13139-3

**Published:** 2022-04-11

**Authors:** Selema Akuiyibo, Jennifer Anyanti, Babatunde Amoo, Dennis Aizobu, Omokhudu Idogho

**Affiliations:** Society for Family Health, Abuja, Nigeria

**Keywords:** Hypertension, Behaviour change communication, Diabetes, Health promotion

## Abstract

**Background:**

Behaviour change communication is a proven health communication strategy among used in promoting changes in knowledge, attitudes, beliefs, and behaviours’, especially for communicable diseases. Few studies have been conducted on its effectiveness for non-communicable diseases prevention and control. This study was conducted as an evaluation assessment for a non-communicable disease focused intervention implemented in Imo and Kaduna States, Nigeria.

**Methods:**

A twelve-month long strategic behaviour change communication intervention on hypertension and diabetes was implemented in selected communities across Imo and Kaduna States, Nigeria. This study adopted a quasi-experimental design approach among adult residents aged at least 35 years to assess the effectiveness of the intervention. Data was collected at baseline (prior to implementation of the interventions) and at the endline; among study and control groups. A uniform study tool was used to collect information on awareness & lifestyle related factors for Hypertension & Diabetes.

**Results:**

The awareness of hypertension was 98.9% among the respondents in the study group compared to 94.4% among the baseline respondents (χ^2^ = 20.276, *p* < 0.001). The history of blood pressure check was recorded among 86.8% of the study group compared to 79.0% of the baseline group (χ^2^ = 20.27, p < 0.001). In the last 6 months prior to the study, 71.9% of the study group compared to 30.6% of the baseline group (χ^2^ = 243.34, *p* = 0.002) had blood glucose check at least once. Daily alcohol consumers make up 36.8% of the baseline respondents, compared to 22.6% in the study group (χ^2^ = 33.84, p < 0.001) and 30.6% of those in the control group compared to the 22.6% of the study group (χ^2^ = 9.23, *p* = 0.002). The mean (± SD) knowledge score on hypertension and diabetes was 18.12 (± 8.36) among the study group compared to 11.84 (± 6.90) among the baseline group (t = 15.29, *p* < 0.001), and compared to 10.97 (± 8.79) among the control group (t = 13.08, *p* < 0.001).

**Conclusion:**

Significant changes in lifestyle practices, knowledge of hypertension and diabetes and risk perception was observed following the implementation of community-based behaviour change communication interventions. There is a need to increased access to health education and promotion interventions for non-communicable diseases.

## Background

Cardiovascular diseases (CVDs) are the leading cause of deaths globally; responsible for almost 18 million deaths every year [1]. People with hypertension and diabetes have been identified, to be at high cardiovascular risk. Hypertension alone accounts for about half of global CVD morbidity and mortality [2]. By 2025, it is estimated that a third of the world population will be hypertensive, with increasing prevalence in developing nations [3]. Between 2019 and 2020, more than 100 million new persons were estimated to develop diabetes [4]. Although the prevalence of both hypertension and diabetes are higher among the older population, reports have shown increasing risk among younger age group [4, 5]. In Nigeria, the prevalence of hypertension ranges between 27 to 38% among adult populations [6, 7] while around 4% are estimated to be diabetic with higher diabetes prevalence reported in some regions [8, 9].

Generally, four major behavioural risk factors contribute significantly to the high risk of hypertension and diabetes among populations. They include lifestyle related practices such as tobacco use, unhealthy diet and obesity, physical inactivity, and harmful use of alcohol. Knowledge of these risk factors is important in promoting the adopting of positive lifestyle practices. For instance, lifestyle modification has been reported to have the potential to reduce the risk of diabetes by as high as 70% [10]. In addition, early detection of hypertension and diabetes through routine screening practices, counselling and adequate medication adherence among already diagnosed individuals are also effective strategies in preventing and controlling blood glucose and pressure levels respectively [11, 12]

Misconceptions and poor knowledge about hypertension and diabetes still exists among Nigerians [13–16]. Also, the prevalence of unhealthy lifestyle practices in the country are high; 25% of Nigerians are not physically active, 13% practice harmful alcohol consumption and 6% of the population are cigarette smokers [17], with higher proportions reported in a study conducted in the eastern part of the country [18]. Behaviour change communication (BCC) is a strategic and integrated communication process involving a mix of advocacy and social mobilisation to influence and sustain changes in social norms, attitude, values, knowledge and behaviours across different levels in the society such as the individual, household, community and national levels. Specifically, BCC has been proven to be an effective intervention in preventing and reducing the risk of communicable diseases such as Malaria, HIV/AIDS and Covid-19 [19–23].

Since the risk factors for hypertension and diabetes include unhealthy lifestyle practices, BCC could be an effective strategy to improve knowledge and facilitate attitudinal and behavioural change among individuals in typical Nigerian communities. Only a few studies have documented the effectiveness of BCC interventions in the prevention, control and management of NCDs such as hypertension and diabetes. In this study, we analysed the effectiveness of an eight-month long BCC intervention in selected communities of the Access-N project of Society for Family Health across Imo and Kaduna States. Findings from this study may provide an evidence-base for the role of BCC in non-communicable diseases programming and inform decision-making for community level strategies towards reducing the risk of such diseases.

## Methods

### Study area

Imo and Kaduna States are in the south-eastern and north-western regions of Nigeria, respectively. The two states have an estimated population of 13.6 million inhabitants in 2016. Kaduna State is made up of 23 administrative units referred to as local government areas (LGAs) and Imo State, on the other hand, consists of 27 LGAs. According to the National Health Facility Registry, there are 1,197 health facilities in Imo State 43% of which are privately owned. However, only about 23% of the 1419 health facilities in Kaduna State are owned by private individuals.

In 2020, Society for Family Health implemented the “Improving Access to Non-communicable diseases” (Access-N) project over a twelve-month period. Access-N Project focused on providing strategic behaviour change communication intervention which aims to empower communities to make informed and healthy choices, increase disease knowledge, improve care seeking behaviour, and facilitate access and linkage to available treatment options, products and services working in the private and public health sectors for two key NCDs namely – cardiovascular disease (hypertension) and type 2 diabetes in Imo and Kaduna States of Nigeria. The project covered a total of fourteen (14) Local Government areas (LGAs), seven (7) LGAs, in each of the implementing states. In Imo state, Owerri North, Aboh Mbaise, Obowo, Oru West, Oguta, Njaba and Ngor Okpala LGAs were reached with Access-N interventions while in Kaduna state, Kaduna North, Kaduna South, Sabon Gari, Igabi, Zaria, Chikun and Kachia LGAs were reached.

### The intervention

Access-N intervention empowered communities with hypertension and diabetes information, the information on determinants of these conditions, prevention information and available treatment options. The project employed an innovative and integrated demand generation approach in selected communities in the two States through Interpersonal Communication Agents (IPCAs) trained to penetrate communities and create awareness, increase knowledge and facilitate better understanding of the promoted NCDs (hypertension and diabetes). In each state, IPCAs conducted behaviour change communications through one-on-one, group and outreach sessions, to address myths and misconceptions and barriers such as low risk perceptions that hinder desired behaviour change related to hypertension and diabetes risks. Contacts with perceived risks were referred for further education and diagnoses at selected health facilities within the communities.

The trained agents point individuals to service delivery points where treatment services and products can be accessed. They also conducted outreaches in these communities to increase uptake of services and products. Outreaches were conducted at least once a month in each of the seven implementing LGAs. The activities during the outreaches included awareness creation, education and sensitization, health talk, screening of community members for blood glucose and blood pressure and referrals to the nearest health facility. The primary aim of the outreach was to increase disease awareness as well as increase demand for NCD services at health facilities.

### Study design

The study adopted a quasi-experimental design approach among randomly selected adult residents aged at least 35 years in selected Access-N implementing communities and control communities in Imo and Kaduna states. The study was conducted at baseline prior to implementation of Access-N interventions and at the end of the project intervention. In addition, a control group which comprised of individuals resident in communities which were not covered by the Access-N project and who were not exposed to the project’s interventions, was included in the study. The inclusion of the control group was to adjust for the effect of time and exposure to hypertension and diabetes information through other sources asides the Access-N interventions between the baseline and endline assessment periods. The findings from the baseline survey has earlier been independently published [13].

### Study population

The study was conducted among adult residents aged at least 35 years in selected communities of the seven (7) implementing LGAs in Imo and Kaduna States prior to the implementation of the project interventions (referred to as baseline group). Eight months following the wrap up of the Access-N project interventions, an endline assessment of the interventions was conducted in the project communities among residents who were exposed to any of the project interventions over the course of implementation (referred to as study or endline group) and also among individuals who were resident in communities other than the those reached by the project interventions but are within the implementing LGAs of the project (referred to as Control group).

### Inclusion criteria

Imo and Kaduna states resident aged 35 years and above from whom informed consent was obtained and who are willing to participate in the study.

### Sample size estimation

The minimum sample size for this study was calculated as follows:

$$\mathrm{N}\hspace{0.17em}=\hspace{0.17em}\frac{{\left(Z\right)}^{2}p(1-p)}{{d}^{2}}$$

Where N is the sample size, Z is the level of significance that corresponds to the 95% confidence level which is 1.96, p is the prevalence of hypertension in Nigeria, and d is the tolerance error (0.05). The awareness rate of hypertension among adults 18 years and above in Nigeria is 17.4% [6].

N = 1.96^2^ × 0.174 × 0.826 / 0.05^2^.

N = 220.85.

Adjusting for a 10% Non-response rate.

N_new_ = n / (1 – 0.10).

 = 220.85 / 0.9

 = 245.4 ≈ 246.

Sample size is therefore estimated as 246 in each state.

Thus, the estimated minimum sample size is 492 for both states at each phase of data collection.

### Sampling technique

At baseline and end line, the selection of community residents was done using multi-stage sampling method as described below:

### Stage 1: Selection of primary sampling unit (PSU)

For this study, the fourteen (14) Access-N LGAs, seven (7) from each state were used as the Primary Sampling Units. The selected LGAs for this survey are as follows; In Imo state; Owerri North, Aboh Mbaise, Ngor Okpala, Oru West, Obowo, Oguta, and Njaba while in Kaduna State they include, Kaduna North, Kaduna South, Sabon Gari, Zaria, Igabi, Chikun and Kachia LGAs.

### Stage 2: Selection of catchment area

A catchment area is defined as an area in the PSU (LGA) where the project’s intervention was implemented. The project adopted a hub and spoke model for participating health facilities where a hub was a facility that can provide comprehensive hypertension and type 2 diabetes services and a spoke was a lower cadre facility that could provide screening services and facilitate referrals to the hubs.

As designed by the Access-N project team, each LGA was made up of a maximum of 5 partner facilities (1 hub and 4 spokes) already identified and selected by the project team. The hub was in a central location surrounded by spokes within 5 km distance of the hub. Communities, dwelling structures around these facilities make up the catchment area for this study.

### Stage 3: Selection of dwelling structure/households

The Access-N participating facilities were identified as the starting point in each LGA. A direction (North, South, West, or East) of the starting point was randomly selected and data collection took place in that direction until the last house on that street was reached and then data collection continued in the next street until the target sample size in the catchment area of the LGA was met.

The day’s code was used to determine the first dwelling structure to begin with. A dwelling structure is a distinct floor of a residential building. Hence a single storied building comprised of two dwelling structures while a bungalow contains a single dwelling structure.

The first dwelling structure was selected using the day’s code method (the summation of the digits of the day’s date to get a single digit e.g. on the first day of the month, the day code is 01, 0 + 1 = 1, the first house). The corresponding house will be the first dwelling structure where an interview will be conducted. If the structure has more than one household (a group of people who usually live together), the household to begin from was randomly selected by balloting.

Every fifth house was visited in a low-density area and ten buildings in a densely populated area to ensure wide coverage of the catchment area. Once a successful call (interview) was made, the interviewer left the entire dwelling structure completely and observed the required sampling gap. This meant that, only one household in a dwelling structure was selected and one respondent in a household, no matter the size of the dwelling structure. This was because the households were homogenous in nature and also to ensure wider spread of interviews in each catchment area and PSU.

### Stage 4: Selection of respondents

Not more than two eligible respondents (male or female aged 35 years and above) were randomly interviewed depending on the population density of a household except in a clustered location. While no gender quotas were set, calls were alternated between males and female to prevent a skew towards any gender.

In a clustered location, such as a marketplace or a restaurant/bar, 10% of the total estimated population of eligible respondents was randomly selected and interviewed.

### Study tool

A uniform study tool was used among the three groups interviewed in this study. Data was collected using self-administered, semi-structured questionnaires and through personal interview for respondents that are not literate. The questionnaires were designed in English language for ease of administration for literate respondents or interpreted to the respondent’s indigenous language and completed with the aid of the interviewer for those who were not able to complete it by themselves. The knowledge, awareness and lifestyle related factors for hypertension and diabetes questions were adapted from WHO STEPS survey tool [24].

The questionnaire was divided into four broad sections which included: A) Background Characteristics of respondent, B) Awareness, Lifestyle-related & screening practices for Hypertension & Diabetes C) Awareness of risk factors, complication and prevention measures for hypertension and diabetes and D) Medication Adherence among Hypertension and Diabetes patients. Section B included questions on smoking practices, alcohol consumption, exercising, fruits & added salt consumption and screening for blood pressure and blood glucose. In section C, the respondents were asked to identify the risk factors, possible complications, and the measures for preventing raised blood glucose and pressure levels while for section D, the respondents who have been previously diagnosed for hypertension and diabetes were asked about their medication adherence for their prescribed anti-hypertensive and anti-diabetic medications.

### Data management and analysis

Questionnaires were checked for errors or omissions at the end of each day and data was subsequently entered and stored in a password secured computer to ensure confidentiality. Following review of the data, only individuals with adequate responses to the questions in the study tool were included in the analysis (hence the difference in sample size across the study groups). SPSS version 20.0 was used for the analysis of the data collected. The frequency distribution and proportions of all the variables in the broad sections listed above were determined across study groups. Chi-square test was used to compare proportions and investigate statistical significance between the baseline and study groups, and the study and control groups for attitude and practices related questions while an independent T-test was used to compare the difference in knowledge across the groups. P-values less than 0.05 were considered as statistically significant.

The respondents who knew the meaning of hypertension and/or diabetes were classified as being aware of either or both two conditions while those who had checked their blood pressure and/or blood glucose levels were regarded to have a history of check for either or both conditions. Correct responses to the each of the items in Section D of the study tool were assigned a score of 1 while incorrect responses were assigned a score of 0. In total, the maximum obtainable score for hypertension knowledge was 17 as there were six items on hypertension risk factors, six items on hypertension prevention measures and five items on hypertension complications. On the other hand, the maximum obtainable score for diabetes knowledge was 18 as there were six question each on diabetes risk factors, prevention measure and complications.

## Ethical approval

Ethical approval was obtained from the Institutional Review Board of the Nigerian Institute of Medical Research (Protocol No: IRB/20/015). Participation was voluntary after each respondent had received detailed information on the purpose of the study and a written informed consent was obtained before questionnaires were administered. In addition, informed consent was obtained from a legal guardian for involved illiterate participants in the study.

## Results

A total of 824 respondents were included in the baseline group while 624 and 500 respondents were included in the study and control groups, respectively. Among the baseline, study and control groups, comparable proportions of the respondents were distributed across characteristics. More females and younger respondents were in each group. Married individuals, Christians and those with a secondary education level were spread across the baseline, study and control groups as shown in Table 1.

**Table 1:** Background characteristics of respondents

Variable	Baseline (%)	Study group (%)	Control (%)
**Age (Years)**			
35 – 44	44.5	33.4	36.2
45 – 54	29.1	29.7	28.0
55 – 64	17.2	23.1	20.4
65 & above	9.1	13.8	15.4
**Gender**			
Male	49.2	42.9	47.2
Female	50.8	57.1	52.8
**State of Residence**			
Imo	57.2	53.1	55.4
Kaduna	42.8	46.9	44.6
**Marital Status**			
Single	10.6	8.1	10.0
Married	79.0	78.7	75.2
Divorced	2.1	1.1	1.4
Widowed	7.9	10.5	11.0
Separated	0.5	1.6	2.4
**Religion**			
Christianity	67.8	53.1	60.6
Islam	31.6	39.3	34.0
Others	0.6	7.6	5.4
**Highest level of Education**			
No Education	10.6	11.3	14.8
Primary	19.1	16.9	18.6
Secondary	38.8	46.9	41.6
Tertiary	29.4	25.0	25.0
Qur’anic school	2.2	0.0	0.0
**Employment Status**			
Unemployed	15.2	27.7	25.0
Employed	84.3	72.3	75.0
**Average Monthly Income (Naira)**			
Below N50,000	67.2	74.7	72.2
N50,000—< N100,000	27.4	22.7	26.2
N100,000—< N200,000	4.6	2.1	1.6
N200,000 & Above	0.7	0.5	0.0
**TOTAL**	**824**	**629**	**500**

### Awareness and screening profile for hypertension and diabetes

The awareness of hypertension was 98.9% among the respondents in the study group compared to 94.4% among the baseline respondents (χ^2^ = 20.27, *p* < 0.001). Among the control group, 84.8% were aware of hypertension while only 81.6% of them had ever heard of diabetes (χ^2^ = 81.60, *p*-value < 0.001) as shown in Table 2. Awareness of diabetes was 90.5% among the baseline group compared to 97.1% in the endline group (χ^2^ = 25.22, *p* < 0.001). Also, the history of blood pressure check was recorded among 86.8% of the study group compared to 79.0% of the baseline group (χ^2^ = 14.95, *p* < 0.001). In the last 6 months prior to the study, 452 (71.9%) of the study group compared to 252 (30.6%) of the baseline group (χ^2^ = 243.34, *p* < 0.001) had blood glucose check at least once.

**Table 2:** Awareness and screening profile for hypertension and diabetes

Variable	Baseline group (%)	Study group (%)	χ^2^ (*p*-value^1^)	Study group (%)	Control group (%)	χ^2^ (p-value^2^)
Awareness of hypertension	778 (94.4)	622 (98.9)	20.27 (< 0.001)	622 (98.9)	424 (84.8)	81.60 (< 0.001)
Awareness of diabetes	746 (90.5)	611 (97.1)	25.22 (< 0.001)	611 (97.1)	408 (81.9)	76.36 (< 0.001)
History of blood pressure check	651 (79.0)	546 (86.8)	14.95 (< 0.001)	546 (86.8)	365 (73.0)	34.07 (< 0.001)
History of blood glucose check	471 (57.2)	538 (85.5)	135. 32 (< 0.001)	538 (85.5)	389 (77.8)	11.34 (0.001)
Last blood pressure check within 6 months	458 (55.6)	499 (79.3)	89.49 (< 0.001)	499 (79.3)	261 (52.2)	93.21 (< 0.001)
Last blood glucose check within 6 months	252 (30.6)	452 (71.9)	243. 34 (< 0.001)	452 (71.9)	338 (68.4)	2.27 (0.132)

^*^p-value^1^—showing Chi-square test (**χ**^**2**^) comparison significance between baseline and endline groups

^*^p-value^2^—showing Chi-square test (**χ**^**2**^) comparison significance between endline and control groups

### Hypertension and diabetes related risk and prevention practices

Daily alcohol consumers were 303 (36.8%) among the baseline respondents, compared to 142 (22.6%) in the study group (χ^2^ = 33.82, *p* < 0.001) and 152 (30.6%) of those in the control group compared to 142 respondents in the endline group (χ^2^ = 9.23, *p* = 0.022). Among the study respondents, daily exercisers made up 73.2% compared to 74.2% of the baseline respondents (χ^2^ = 0.18, *p* = 0.672) while 64.2% of the control groups were daily exercisers (Table 3). Daily fruit consumers were 7.9% in the baseline group compared to 20.8% in the study group (χ^2^ = 51.17, *p* < 0.001) and 15.2% in the control group compared to the study group (χ^2^ = 5.89, *p* = 0.015).

**Table 3:** Hypertension and diabetes related risk and prevention practices

Variable	Baseline group (%)	Study group (%)	χ^2^ (*p*-value^1^)	Endline (%)	Control (%)	χ^2^ (*p*-value^2^)
Daily alcohol consumers	303 (36.8)	142 (22.6)	33.84 (< 0.001)	142 (22.6)	152 (30.6)	9.23 (0.002)
Daily smokers	66 (8.0)	39 (6.2)	1.74 (0.187)	39 (6.2)	58 (11.6)	10.4 (0.001)
Daily exercisers	611 (74.2)	458 (73.2)	0.18 (0.672)	458 (73.2)	319 (64.2)	10.48 (0.001)
Daily fruit consumers	65 (7.9)	131 (20.8)	51.17 (< 0.001)	131 (20.8)	76 (15.2)	5.89 (0.015)
Occasional consumers of food with added salt	174 (21.1)	98 (15.6)	7.19 (0.007)	98 (15.6)	497 (28.6)	27.51 (< 0.001)

^*^p-value^1^—showing Chi-square test (**χ**^**2**^) comparison significance between baseline and endline groups

^*^p-value^2^—showing Chi-square test (**χ**^**2**^) comparison significance between endline and control groups

### Knowledge of risk factors, complications and preventive measures for hypertension and diabetes

The mean knowledge score (± SD) on hypertension risk factors was 3.18 (± 1.82) among the study group compared to 1.85 (± 1.41) among the baseline group (t = 15.13, *p*-value < 0.001) and compared to a mean score of 1.90 (± 1.85) among the control group (t = 11.66, *p*-value < 0.001) (Table 4). Similarly, the mean knowledge score on diabetes complication was 2.85 (± 1.68) among the study group compared to 2.12 (± 1.55) among the baseline group (t = 8.54, *p* = 0.001) and compared to a mean score of 1.80 (± 1.55) among the control group (t = 10.94, *p* < 0.001). The overall mean knowledge score for hypertension and diabetes was 18.12 (± 8.36) among the study group compared to 11.84 (± 6.90) among the baseline group (t = 15.29, *p* < 0.001) and compared to 10.97 (± 8.79) among the control group (t = 13.08, *p* < 0.001).

**Table 4:** Respondents knowledge ofr factors, complications and preventive measures for hypertension and diabetes

**Score**	**Baseline group** Mean ± SD	**Study group** Mean ± SD	**T-test (*p*-value**^**1**^**)**	**Study group** Mean ± SD	**Control group** Mean ± SD	**T-test (*p*-value**^**2**^**)**
Hypertension risk factors	1.85 ± 1.41	3.18 ± 1.82	15.13 (< 0.001)	3.18 ± 1.82	1.90 ± 1.85	11.66 (< 0.001)
Diabetes risk factors	1.52 ± 1.18	2.82 ± 1.80	15.77 (< 0.001)	2.82 ± 1.80	1.62 ± 1.71	11.43 (< 0.001)
Hypertension complications	2.03 ± 1.24	2.49 ± 1.41	6.45 (0.001)	2.49 ± 1.41	1.68 ± 1.27	10.08 (< 0.001)
Diabetes complications	2.12 ± 1.55	2.85 ± 1.68	8.54 (< 0.001)	2.85 ± 1.68	1.80 ± 1.55	10.94 (< 0.001)
Hypertension prevention and management	2.36 ± 1.62	3.46 ± 1.82	12.00 (< 0.001)	3.46 ± 1.82	2.07 ± 1.82	12.84 (< 0.001)
Diabetes prevention and management	1.95 ± 1.40	3.31 ± 1.62	16.64 (< 0.001)	3.31 ± 1.62	2.02 ± 1.59	13.41 (< 0.001)
**Total Hypertension and Diabetes Knowledge Score**	**11.84 ± 6.90**	**18.12 ± 8.36**	**15.29 (< 0.001)**	**18.12 ± 8.36**	**10.97 ± 8.79**	**13.08 (< 0.001)**

### Medication Adherence among Hypertension and Diabetes Patients

**Fig. 1:**
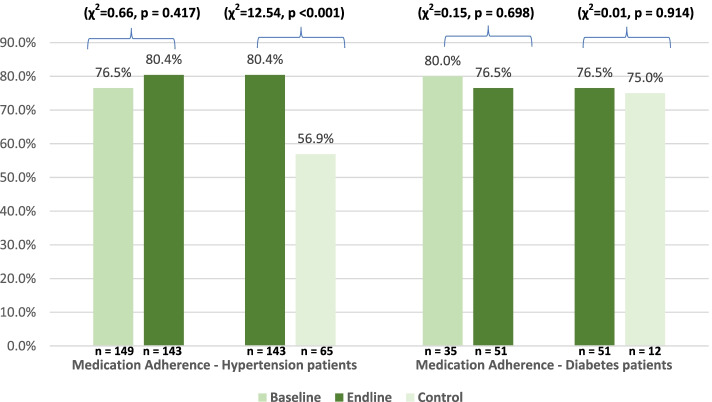
Medication Adherence among Persons diagnosed with Hypertension and Diabetes

Among persons diagnosed with hypertension, 80.4% in the endline group compared to 76.5% in the baseline and 56.9% in the control groups respectively adhere to their medication as prescribed by a healthcare provider. On the other hand, 76.5% of persons diagnosed with diabetes in the endline group compared to 80.0% in the baseline and 75.0% in the control groups adhere to their diabetes medication (Fig. 1).

## Discussion

The Access-N project’s BCC strategy was designed based on the health belief model, socio-cognitive and the diffusion of innovation theories. Our findings generally showed that the awareness of both hypertension and diabetes were significantly higher among the study group compared to both baseline and control groups. As observed in this study (especially among the baseline and control groups), and in other studies, the level of hypertension and diabetes awareness in Nigeria is quite high. In spite of this, the BCC strategy adopted by the Access-N project further contributed to a notable increase in the level of hypertension and diabetes awareness among the study group. Health education in the form of awareness creation for hypertension and diabetes has proven to improve screening service uptake [25]. Similarly in this study, the observed higher awareness of both conditions among the study groups could also be attributed to the increase in the proportion of the respondents who have a history of blood pressure or glucose check in the study group compared to the other groups.

The health belief model theory posits that the self-perceived threat or susceptibility to a health risk will motivate adoption of positive preventative action [26]. Through the BCC strategies of the Access-N project, persons reached by the project were able to properly assess the health implications of some of their lifestyle practices that could predispose them to risks of developing high blood pressure and/or high glucose levels. This further led to the gradual adoption of positive lifestyle behaviour such as increased fruit intake and reduced negative practices such as daily alcohol intake, smoking, etc., observed among the intervention group. Decreases in exercising could be attributed to the COVID19 pandemic which could have reduced the frequency and numbers of outdoor activities in the study locations due to social distancing measures. Also, decreases in fruit intakes could be attributed to lack of purchasing power as a result of inflation which worsened because of the COVID-19 pandemic. Although, the goal of public health transcends the initiation of positive behaviours alone, it further seeks to maintain such behaviours. The initiation of these behaviours within this short period of implementing Access-N intervention shows the potentials for self-efficacy and further suggests that a continued exposure to BCC intervention would be helpful in ensuring that such behaviours are maintained.

At the core of the Access-N BCC strategy is health education on hypertension and diabetes risk factors, preventive measures, and complications. Research has shown that health education intervention on hypertension-related knowledge is effective in improving knowledge about the condition and influencing adoption of self-care practices [27]. In this study, we observed also remarkable increase in hypertension knowledge (as high as 30% for diabetes risk factors) among the study group when compared to the other groups. Whilst knowledge may not be sufficient to result in behaviour change, it is a critical component in motivating behaviour change. Change in behaviour is better assured if health education targets personal relevance [28]. The adopted BCC strategy by the Access-N did not only aimed to improve knowledge, it also focused on personal risks and likely complications that could result from hypertension and diabetes. Thus, the increased knowledge observed among the study group could be further attributed to the increased adoption of some positive behaviours observed in this study.

Behaviours are formed as a result of interactions between individual characteristics, and other social, political and economic factors. Thus, a holistic approach is required in driving behaviour change [29, 30]. In this study, we observed that among the hypertension and diabetes patients, BCC was not significantly effective in fostering medication adherence, especially among the diabetes patients. Other non-knowledge related factors such as perceived feeling of wellness, high cost of prescribed medications, perceived medication efficacy, could be responsible for non-medication adherence [31]. In choosing the right intervention for addressing non-adherence, there is a need to understand major causal factors. This will assist in designing motivations for adherence using appropriate behaviour change model or product design interventions.

### Study limitations

Our study is not without limitations. The respondents across groups, (specifically the baseline and study groups) were not matched and had different sample sizes. Thus, confounding variables might have contributed to some of the changes observed in this study. However, we ensured that the distribution of characteristics among the respondents across groups are very similar. Also, a control group was not included during the baseline assessment. The inclusion of a baseline control group would have better explained comparisons and attributions of the observed changes to the intervention. However, we independently made comparisons between the study and both the baseline and the control groups, respectively in order to adjust for the effect of any other exposure that could be responsible for the outcomes assessed. Despite these shortcomings, we believe that the findings from this study are valid and could be useful to make recommendations for hypertension and diabetes prevention and control.

## Conclusions

Strategic behaviour change communication was found to be quite effective in promoting the adopting of positive lifestyle practices essential for hypertension and diabetes control. In addition, significant improvement in knowledge was also observed as a result of the intervention. In order to reduce the burden of hypertension and diabetes in the population, there is a need to increase and improve access to quality information on hypertension and diabetes prevention, treatment and control among adults through a mix of cost-effective community-based health promotion strategies.

## Data Availability

All available data can be obtained by contacting the corresponding author. Access to anonymised data may be granted following review.
